# Toward synthetic plant development

**DOI:** 10.1093/plphys/kiab568

**Published:** 2021-12-14

**Authors:** Jennifer A N Brophy

**Affiliations:** Department of Bioengineering, Stanford University, Stanford, California 94305, USA

## Abstract

The ability to engineer plant form will enable the production of novel agricultural products designed to tolerate extreme stresses, boost yield, reduce waste, and improve manufacturing practices. While historically, plants were altered through breeding to change their size or shape, advances in our understanding of plant development and our ability to genetically engineer complex eukaryotes are leading to the direct engineering of plant structure. In this review, I highlight the central role of auxin in plant development and the synthetic biology approaches that could be used to turn auxin-response regulators into powerful tools for modifying plant form. I hypothesize that recoded, gain-of-function auxin response proteins combined with synthetic regulation could be used to override endogenous auxin signaling and control plant structure. I also argue that auxin-response regulators are key to engineering development in nonmodel plants and that single-cell -omics techniques will be essential for characterizing and modifying auxin response in these plants. Collectively, advances in synthetic biology, single-cell -omics, and our understanding of the molecular mechanisms underpinning development have set the stage for a new era in the engineering of plant structure.

## Teaser

Recent advances in our understanding of plant development and ability to control gene expression in plants may enable a new strategy for engineering plant form and function.

## Introduction

**Figure 1:**
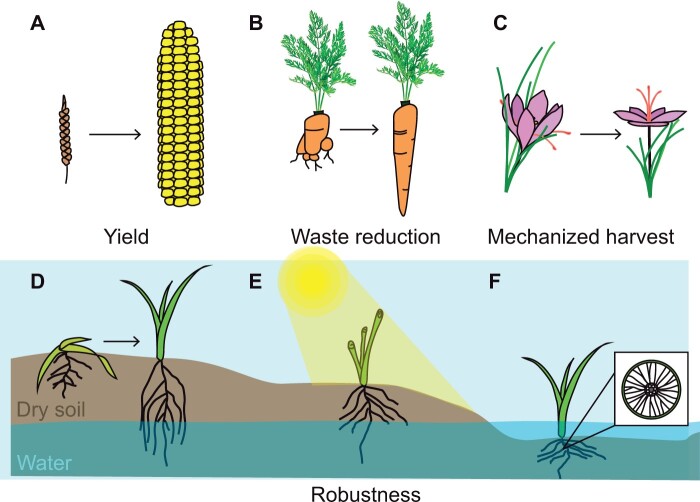
Potential benefits of engineering plant structure. Here are several hypothetical ways that plant structure could be engineered to enhance our agricultural systems. A, Plants could be engineered to increase yield by changing the size and number of seeds or fruits produced. B, Growth programs could be modified to ensure that all produce meets market size and shape standards. C, Plants could be made compatible for automated harvest. Here, saffron flowers are modified to present their stigma and styles to a machine for harvest. Other solutions may involve encasing delicate produce in tough cellulosic structures to prevent damage during harvest. D and E, Structural modifications to improve environmental stress tolerance. D, Roots engineered to reach water in deep layers of soil enable plants to survive prolonged droughts. E, Leaves curled to reduce heat stress from direct sun exposure. F, Air channels (called aerenchyma) introduced to roots to enable survival in flooded soils. Aerenchyma delivers oxygen to submerged tissues in order to prevent hypoxia and tissue death.

Achieving control over plant development will revolutionize agriculture by enabling the design and production of plants with specific forms. Modifying plant shape could streamline food production by increasing yield, reducing waste, and facilitating mechanized harvest. Already, changes in the structures of important food crops, like wheat (*Triticum aestivum*), rice (*Oryza sativa*), and maize (*Zea mays*), have dramatically increased food production (Kaur, 2011; Stitzer and Ross-Ibarra, 2018; Figure 1, A). Similarly, engineering of tomato (*Solanum lycopersicum*) and groundcherry (*Physalis grisea*) structure has yielded varieties with high productivity in vertical farms (Kwon et al., 2020). Engineering plant shape may help reduce farm-level food loss by ensuring all produce meets market size and shape standards (Figure 1, B; USDA, 2021). Excitingly, the ability to redesign plant structure also opens up the possibility of engineering both sides of the plant–machine interface to produce more efficient farms. In such scenarios, plant structure would be modified to produce forms that enable mechanized harvest in order to reduce both labor cost and unharvested food waste (Figure 1, C).

Engineering plant structure may also allow us to produce crops that are robust to climate change. Plants often survive in challenging environments by changing their growth programs to produce forms that enable survival. For example, roots of some drought tolerant plants elongate to reach water in deep layers of soil when the upper layers dry out (Koevoets et al., 2016). In other heat-tolerant plants, leaves may curl in order to reduce direct sun exposure and water loss (Fitter and Hay, 2002). Introducing these tolerizing features into climate-sensitive plants may help our crops survive the environmental challenges that are predicted to negatively impact food production in the coming decades (Kummu et al., 2021; Figure 1, D–F). Field experiments with *DEEPER ROOTING 1* (*DRO1*) and *SUBMERGENCE 1* (*sub1A-1*) overexpressing rice varieties show that modifying root length and stem structure enhance rice survival in drought and flood conditions, respectively (Xu et al., 2006; Uga et al., 2013). A next stage in plant engineering could involve the movement of these features to other sensitive crops, even those that lack *DRO1* and *SUB1* orthologs and cannot be made stress tolerant using the same genetic mutations as rice. In these cases, new methods of engineering form will be essential for introducing robust structural features to sensitive plants.

Although the benefits of engineering plant form are vast, our capacity to reprogram plant growth remains limited. Crop structure is most commonly modified through random mutagenesis and screening, or breeding with cultivars that contain desirable traits. These methods allow researchers to generate changes in structure without knowledge of the underlying genetic mechanisms. Important modern crops, such as the dwarf rice and wheat varieties that enabled the Green Revolution, were produced through breeding nearly 30 years before the *Rht* mutations that lead to gibberellin insensitivity and dwarfism were understood at the molecular level (Peng et al., 1999; Silverstone and Sun, 2000). Though very useful, mutagenesis and breeding can be imprecise and limited in terms of the plant structures that can be created (Burstin et al., 2007; Xie et al., 2018). Thus, there is interest in moving beyond conventional methods to redesign plant structure through genetic engineering (Dangl et al., 2018).

The field of synthetic biology is uniquely positioned to enable a new phase in plant form engineering. Decades of research have revealed many of the molecular mechanisms underpinning the size, shape, and production of plant roots, flowers, leaves, and seeds. Plant development is now known to be controlled by families of transcription factors, whose expression and activity are regulated by hormones, refined by collections of post-transcriptional and post-translational regulators, and complicated by extensive crosstalk between individual components (Alberts et al., 2002; Vanstraelen and Benková, 2012; Chen et al., 2018). At first glance, it is unclear how any individual developmental process could be engineered. Yet, synthetic biologists have developed a method for managing and modifying complex biological processes called refactoring, which could be useful for reprogramming plant development. Refactoring is the process of recoding a biological function by first knocking out all genes involved in a process and then reintroducing them under the control of well characterized synthetic genetic parts, such as promoters, ribosome binding sites, and terminators (Chan et al., 2005). Reintroduced genes are usually “codon randomized” to eliminate internal regulation. The refactored pathways are designed to enable easy swapping of regulatory elements in order to facilitate changes in patterns and dynamics of gene expression. Synthetic regulatory elements are chosen to closely match the activites of the native promoters such that refactored pathways recapitulate the functions of the original pathway, but are much easier to modify. To date, synthetic biologists have been able to refactor bacteriophage to deliver anticancer drugs to tumor cells (Ghosh et al., 2012), nitrogenase gene clusters to boost the nitrogen fixing capabilities of soil bacteria (Temme et al., 2012; Ryu et al., 2020), silent biosynthetic gene clusters to stimulate the production of antibiotics (Shao et al., 2013), and type III secretion systems to move the capacity to secrete protein from one bacterial species to another (Song et al., 2017).

Refactoring plant development will require a unique approach. Extensive crosstalk between developmental processes in plants makes the knockouts that precede traditional refactoring challenging. In this review, I propose a knockout-free refactoring-like approach that uses mutant auxin response regulators to modify the development of otherwise wild-type plants. I hypothesize that gain-of-function auxin response regulators that override native developmental cues could be used to engineer plant structure. Here, I outline a method that synthetic biologists and plant biologists could use to tinker with plant development, and I discuss the benefits and limitations of engineering plant structure using this approach.

## Engineering auxin-insensitive development

**Figure 2:**
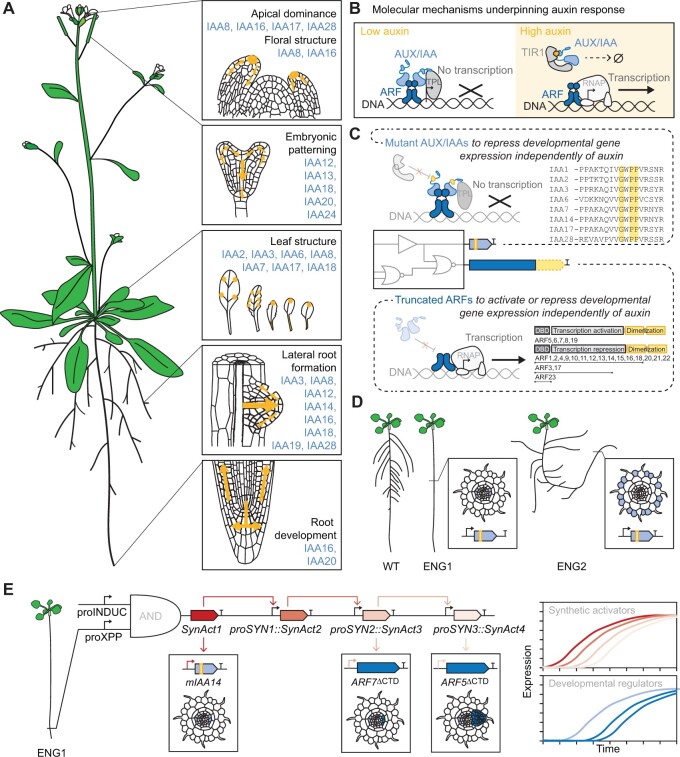
Engineering development through auxin response factors. A, Auxin’s role in Arabidopsis growth and development. Yellow arrows show auxin distribution in various plant tissues during development. Aux/IAAs involved in Arabidopsis organ development listed in blue. *Source*: Illustrations (2020); Luo et al. (2018); and Teale et al. (2006). B, Molecular mechanisms of auxin response. In cells with little to no auxin, ARF transcription factors are bound by Aux/IAAs, which inhibit ARF activity by recruiting the transcriptional repressor TOPLESS (TPL). When the concentration of auxin increases, Aux/IAAs interact with TIR1/AFB auxin co-receptors, which lead to Aux/IAA ubiquitination and degradation. ARFs subsequently become free to regulate the transcription of their target genes. This panel illustrates auxin regulation of an ARF activator; however, members of the ARF protein family can also function as auxin-dependent repressors or auxin-independent transcription factors. C, Mutations that make Aux/IAAs (upper panel) and ARFs (lower panel) insensitive to auxin. Aux/IAA inset shows protein alignment of Aux/IAAs’ highly conserved domain II, which enables auxin-induced degradation. Residues that can be mutated to yield auxin-insensitive Aux/IAAs are highlighted in yellow (inset adapted from Fukaki et al., 2002). ARF inset shows the protein domains present in Arabidopsis ARFs: an N-terminal B3-type DNA binding domain, a variable middle region that functions as a transcriptional activation or repression domain, and a C-terminal Phox and Bem1p (PB1) domain that facilitates homo- and heterodimerization. Auxin-sensitive ARFs are bound by Aux/IAAs through the PB1 domain. Though the majority of Arabidopsis ARFs contain all three domains, some ARFs are naturally auxin-insensitive because they are missing the PB1 domain or contain PB1 domains that do not support Aux/IAA dimerization. Auxin-responsive ARFs can be made insensitive by removing their PB1 domain (yellow) and eliminating ARF-Aux/IAA interaction. Inset adapted from Legrand et al. (2016). D, Hypothetical results of engineering Arabidopsis root structure using mutant Aux/IAAs. WT, wild type; ENG1, plant engineered to have no lateral roots by expressing a mutant Aux/IAA in xylem pole pericycle cells; ENG2, plant engineered to reduce gravitropism by expressing a mutant Aux/IAA in elongating epidermal cells. E, Hypothetical scheme for reintroducing root branching to the ENG1 lateral rootless plant from (D). An AND gate is used to trigger a transcriptional cascade in xylem pole pericycle cells after an inducing signal. The AND gate combines the activity of an inducible promoter (proIND), such as β-estradiol or dexamethasone (Borghi, 2010), and a xylem pole pericycle-specific promoter (proXPP), such as SLAC1 HOMOLOG (SLAH1) (Cubero-Font et al., 2016). Activation of the AND gate leads to the expression of a synthetic activator (SynAct1), which drives the expression of *GATA23* to create LRFCs and a second synthetic activator (SynAct2) to continue the signaling cascade. SynAct2 initiates the expression of SynAct3, which subsequently initiates the expression of engineered ARF7 and ARF19 proteins (ARF7^ΔCTD^ and ARF19^ΔCTD^) to initiate the first steps in lateral root development. SynAct3 continues the cascade by initiating the expression of a fourth synthetic activator (SynAct4). SynAct4 then initiates the expression of ARF5^ΔCTD^ to continue lateral root development. In this synthetic developmental program, the cascade is used to temporally separate the expression of each developmental regulator. Theoretical expression profiles of each gene in the program are shown on the right-hand side of the panel, with lines color coded to match the genes in the schematic. Expression profiles based on previous research building synthetic cascades in bacteria (Hooshangi et al., 2005). SynAct2 is a “silent” step in the cascade because it does not drive expression of a developmental regulator. Inclusion of SynAct2 leads to greater temporal separation between *GATA23* and *ARF7^ΔCTD^/ARF19^ΔCTD^* expression. Other features of the cascade, including activator strength and cooperativity, could also be used to tune expression dynamics. This synthetic program could be used to study the impacts of gene expression dynamics on lateral root development. Since auxin triggers several distinct steps in lateral root development, interpretation of the auxin signal must depend in part of on the state of the receiving cell. Synthetic regulation provides a unique opportunity to analyze the time necessary to establish developmental competence in lateral root precursors and the consequences of initiating downstream processes too early or too late.

The hormone auxin is a key regulator of plant development. Auxin regulates the size and shape of almost every organ in the model plant Arabidopsis (*Arabidopsis thaliana*) (Teale et al., 2006; Luo et al., 2018; Figure 2, A). Auxin concentrations define locations of developmental gene expression that lead to differentiation, division, cell elongation, and senescence. Changes in auxin biosynthesis and degradation can cause severe developmental defects, including the production of sterile flowers, overproliferation of roots, and abnormal embryogenesis (Ploense et al., 2009; Zhao, 2010). Similarly, defects in auxin transport can completely eliminate flower/seed production, alter cotyledon shape and number, and disrupt vascular patterning (Křeček et al., 2009; Adamowski and Friml, 2015). Auxin’s central role in plant development makes it an attractive target for engineering structure.

Key auxin response factors could serve as powerful tools for reprogramming development. Two well-characterized protein families are central to auxin response:AUXIN RESPONSE FACTORS [ARFs] and Aux/IAA (Li et al., 2016; Luo et al., 2018; Figure 2, B). ARFs are transcription factors that bind DNA and control the expression of developmental genes. ARF proteins are typically composed of three domains: an N-terminal B3-type DNA binding domain, a variable middle region that functions as a transcriptional activation or repression domain, and a C-terminal Phox and Bem1p (PB1) domain that facilitates homo- and heterodimerization (Li et al., 2016). In cells with little to no auxin, Aux/IAAs bind ARFs through their PB1 domain and inhibit ARF activity. When the concentration of auxin increases, Aux/IAAs are ubiquitinated and targeted for degradation by TIR1/AFB auxin co-receptors. Auxin-mediated Aux/IAA degradation frees ARFs to regulate the transcription of their target genes. Auxin-responsive ARFs and Aux/IAAs could be could be modified to override auxin signaling by truncating ARFs to remove the C-terminal PB1 domains that mediate ARF-Aux/IAA interaction or by changing amino acids in Aux/IAAs to prevent auxin-dependent degradation (Guilfoyle, 2015; Moss et al., 2015; Figure 2, C). Mutant ARFs and Aux/IAAs could then be used to activate and repress gene expression independently of auxin and engineer development (Rouse et al., 1998; Wang et al., 2005).

Although the canonical auxin response pathway is simple, not all ARFs are regulated by auxin and Aux/IAAs. Several ARFs lack a PB1 domain and current research suggests that these ARFs function as constitutively active transcription factors that fine tune gene expression by competing with auxin-responsive ARFs for DNA binding sites (Lavy et al., 2016; Kato et al., 2020). Genetic engineers may use these naturally auxin-insensitive ARFs to tune the expression of developmental genes by expressing them in specific tissues or at specific levels to interfere with the activity of mutant ARF proteins.

In addition to the canonical auxin response pathway, ARF and Aux/IAA activity is regulated by post-transcriptional and post-translational regulators, including microRNAs, small interfering RNAs, upstream open reading frames, kinases, phosphatases, and SUMOlases. These regulators that are controlled by nonauxin signals, such as other plant hormones and environmental stimuli, and typically fine-tune the auxin response (Mallory et al., 2005; Teotia et al., 2008; Rosado et al., 2012; Li et al., 2016; Orosa-Puente et al., 2018). To sever the interactions between these regulators and ARFs/Aux/IAAs, the DNA that encodes for ARF and Aux/IAA could be recoded. In a recent paper, researchers studying ARFs in *Marchantia polymorpha* introduced a silent mutation to *MpARF3* to prevent microRNA regulation (Kato et al., 2020). Taken one step further, the entire *ARF* and *Aux/IAA* genes could be recoded to remove regulation by noncoding RNAs. Excitingly, codon randomizing entire genes has the potential to remove regulation by multiple noncoding RNAs simultaneously, even those that have not yet been discovered or characterized. In bacteria, codon randomization has been used to remove embedded cis regulatory elements from genes (Chan et al., 2005). Thus, in addition to preventing recognition by noncoding RNAs, recoding may further insulate *ARF* and *Aux/IAA* genes from native regulation by removing cis regulatory elements, such as transcription factor binding sites or regulated splice sites. In previous refactoring efforts, codon randomized genes were computationally screened to identify any unintentionally introduced undesirable sequences, such as restriction, methylation, and transposon sites (Chan et al., 2005; Temme et al., 2012). If applied in plants, recoding should involve a similar screening process to prevent the introduction of unintended cis regulatory elements in recoded genes.

At the protein level, nonsynonymous point mutations may further insulate ARF and Aux/IAA activity from native developmental cues. Scientists have frequently substitute phosphorylated or SUMOylated residues for other amino acids in order to mimic or prevent post translational modifications. For example, a recent study of root branching in Arabidopsis replaced key lysine residues in AtARF7 with arginines to block regulation by the small ubiquitin-like modifier (SUMO) protein (Orosa-Puente et al., 2018). Using a similar approach, genetic engineers may be able to decouple ARF and Aux/IAA activity from regulation by post-translational modifying enzymes. Although our understanding of ARF and Aux/IAA regulation is likely incomplete, recoding at both the DNA and amino acid level is an approach that should be able to insulate ARF and Aux/IAA activity from many of the known nonauxin signals. As genetic engineers begin to use recoded ARFs and Aux/IAAs to control gene expression in planta, they may discover that the proteins’ activities are still modified by auxin to an extent that interferes with their developmental engineering goals. In these scenarios, it may be possible to use the recoded ARF and Aux/IAA genes as tools to elucidate additional mechanisms through which auxin can modify ARF and Aux/IAA activity. By reducing the number of mechanisms by which auxin is able to regulate ARF and Aux/IAA activity, recoded genes may streamline mechanistic studies. Any additional forms of auxin regulation may require the development of new approaches for decoupling ARF and Aux/IAA activity from auxin signaling.

Once decoupled from auxin signaling, ARFs’ and Aux/IAAs’ impact on development should be defined by their expression levels and patterns across plant tissues. Arabidopsis plants containing auxin-independent gain-of-function alleles of Aux/IAAs are usually defective in multiple plant structures because the mutant proteins are expressed in many cell types throughout the plant. For example, *IAA14* is expressed in almost all organs in Arabidopsis, including roots, leaves, stems, and flowers (Abel and Theologis, 1996). Therefore, an auxin-insensitive mutant allele of *IAA14* called *SOLITARY ROOT* (*slr-1*) impacts the development of several plant organs. Not only does the mutation eliminate lateral roots and root hairs, it also reduces primary root growth, inhibits gravitropism, and reduces shoot size (Fukaki et al., 2002). In contrast, tissue-specific expression of auxin-insensitive Aux/IAAs can have a more specific impact on development and have been used as tools to elucidate the role of auxin signaling in specific cell types. For example, an auxin-insensitive mutant version of IAA17 (AUXIN INSENSITIVE3) was expressed in lateral root cap and expanding epidermal cells to identify tissues in which auxin response is important for root gravitropism (Swarup et al., 2005). Given auxin’s central role in development, the expression of auxin-insensitive mutants may have pleiotropic effects in spite of using tissue-specific promoters. Thus, the magnitude and timing of ARF and Aux/IAA expression will likely need to be precisely regulated in order to make targeted changes in development. It may also be necessary to express collections of ARF and Aux/IAA proteins that antagonize each-others’ activity in order to initiate specific developmental processes and reduce pleiotropic effects.

To engineer plant structure, synthetic regulatory programs could be designed and built to express mutant ARFs and Aux/IAAs in patterns that alter the size, shape, placement, or abundance of specific organ types (Figure 2, D). As an example, *slr-1* could be expressed in lateral root founder cells (LRFCs) to prohibit lateral root formation using a founder-cell-specific promoter, such as GATA23 (Fukaki et al., 2002; Rybel et al., 2010). Then mutant ARFs could be used to re-introduce root branching by overriding the IAA-based repression and triggering the expression of appropriate downstream genes (Box 1). Synthetic regulatory programs that recapitulate the spatiotemporal patterns of ARF activity in normal development should resemble wild-type plants, whereas synthetic regulatory programs that deviate from normal expression patterns should alter development. By designing synthetic regulation to express ARF and Aux/IAAs in patterns that alter the placement, structure, and abundance of plant organs, engineers may be able to create plants with new and useful forms.

Box 1: Hypothetical scheme for engineering root branching.

It may be possible to engineer plant structure by expressing auxin-insensitive ARFs and Aux/IAAs in patterns that alter the size, shape or abundance of specific plant organs. Here, we describe a theoretical synthetic developmental program that could be used to generate root branches in the model plant Arabidopsis. This program uses auxin-insensitive ARFs to trigger the expression of developmental genes necessary for lateral root development: Studies show that at least three distinct auxin-gated steps are essential for lateral root development: (1) IAA28-dependent founder cell specification, (2) IAA14-dependent ARF7/ARF19 de-repression, which leads to nuclear migration and asymmetric founder cell divisions, and (3) IAA12-dependent ARF5 de-repression, which leads to root initiation and organogenesis (Smet et al., 2010; Goh et al., 2012). A synthetic program that produces root branches may also be composed of three steps: (1) *GATA23* expression in xylem pole pericycle cells to create LRFCs, (2) *ARF7^ΔCTD^* and *ARF19^ΔCTD^* expression in the synthetically derived LRFCs to initiate nuclear migration and cell division, and (3) *ARF5^ΔCTD^* expression to continue organogenesis. In this synthetic developmental program, the expression of each gene would be regulated by a synthetic genetic “controller” that defines spatiotemporal patterns of expression. For this example, the controller may be a synthetic cascade that is initiated in xylem pole pericycle cells and propagated by a series of synthetic transcription factors that activate expression of each mutant ARF (Figure  2, E; Hooshangi et al., 2005). The synthetic program may also express genes in neighboring root epidermis, cortex and endodermis cells to remodel these tissue layers. Remodeling of root epidermis, cortex, and endodermis tissues is important for lateral root emergence (Vilches-Barro and Maizel, 2015). Thus, the synthetic program may need to coordinate gene expression between cell layers. Synthetic cell–cell signaling has not yet been demonstrated in plants but has been achieved in bacteria, yeast, and mammalian cell lines using diffusible quorum sensing molecules, nitrous oxide, fungal pheromones, and synthetic Notch receptor proteins (Hennig et al., 2015; Toda et al., 2018). Bringing synthetic cell–cell signaling into plants to replace auxin will require the development of new gene expression tools. Ultimately, synthetic genetic controllers that are capable of precisely regulating the temporal and magnitudinal patterns of gene expression in plants should be powerful tools for engineering development.

One drawback of synthetic development is that it requires an in-depth understanding of the molecular mechanisms underpinning a developmental process. Gaps in our understanding will be exposed by synthetic programs that lack key regulators and therefore do not develop as expected. For example, the synthetic lateral root development scheme (Box 1) assumes that GATA23 is sufficient for founder cell establishment, which has been suggested, but not proven (Rybel et al., 2010). Similarly, it assumes that the overexpression of mutant ARF7, ARF19, and ARF5 in xylem pole pericycle cells will be sufficient for progressing lateral root development from initiation to emergence. Although these proteins are essential for lateral root development, additional genes will likely need to be co-expressed to repress some of the ARF7, ARF19, and ARF5 target genes or to initiate processes that are not activated by these ARFs (Okushima et al., 2007; Smet et al., 2010). Careful morphological analysis of engineered roots along with comparative single-cell transcriptomic studies can be performed to troubleshoot nonfunctional synthetic developmental programs. These methods should identify defects in development and additional genes that need to be expressed or repressed to produce functional organs and tissues. In the lateral root example, comparative transcriptomics of LRFCs in wild-type plants and engineered plants expressing GATA23 in xylem pole pericycle cells may help identify genes that are essential for founder cell identity. Though our understanding of plant development is undeniably incomplete and it will take decades longer to comprehensively map the genetic programs that define the size and shape of each plant organ, we can use our current models of plant development to generate testable hypotheses about the structural impacts of overriding auxin response in individual tissues at specific times (Outstanding questions Box). Initial engineering projects may not generate the intended changes in plant structure; however, careful analysis of each engineered plant may fill gaps in our current understanding of development and accelerate the generation of developmental models that can be used to successfully engineer plant form.

Synthetic developmental regulation will require exquisite control over gene expression in plants. ARFs and Aux/IAAs are developmental sledgehammers, with the capacity to alter many different developmental processes simultaneously. To successfully engineer individual aspects of complex plant organs, like roots, leaves, and flowers, the expression patterns of ARFs and Aux/IAAs will need to be carefully controlled. Any leaky misexpression of an ARF or Aux/IAA could cause a synthetic developmental program to fail. Though our ability to precisely control gene expression in plants has historically been limited, advances in our understanding of how to build complex synthetic genetic programs in bacteria and yeast, such as multi-input logic gates (Nielsen et al., 2016), oscillators (Elowitz and Leibler, 2000; Rossi and Dunlop, 2018), transcriptional cascades (Hooshangi et al., 2005), and de novo pattern forming programs (Basu et al., 2005; Sekine et al., 2018), provide roadmaps for engineering synthetic developmental regulation in plants. Researchers have shown that the timing of gene expression can be controlled using synthetic transcriptional cascades of increasing length (Hooshangi et al., 2005) and that amplitude and period of gene expression oscillations can be modified through changes in transcription, translation, and degradation rates of oscillator components (Stricker et al., 2008; Tomazou et al., 2018). Preliminary experiments building similar regulatory programs in bacteria and mammalian cell lines suggests that some of the basic design rules for regulatory circuits may apply in both prokaryotes and eukaryotes (Tigges et al., 2009; Stanton et al., 2014). Plus, new molecular tools for controlling transcription and translation in plants, such as synthetic promoters (Cai et al., 2020; Jores et al., 2021), polyadenylation signals (Felippes et al., 2020; Andreou et al., 2021), and transcription factors (Schaumberg et al., 2016; Belcher et al., 2020), are being developed at break neck speeds. Additional tools that improve our control over gene expression in plants will be needed to create synthetic developmental regulation (Outstanding Questions Box).

## Engineered plants as tools for testing form–function relationships in plants

**Figure 3:**
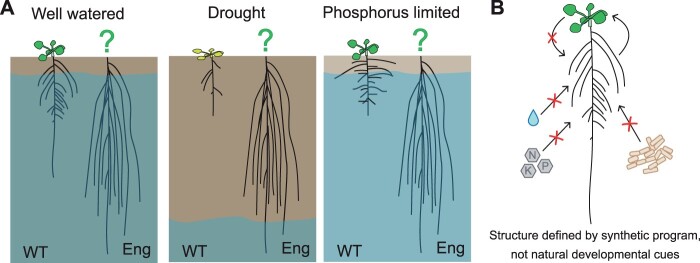
Auxin-insensitive growth as a means of testing the relationships between plant form and function. Potential uses for plants containing synthetic auxin-/environment-independent developmental programs. A, Influence of root structure on plant health could be tested in different environments. Engineered plants (eng) produce the same root system in every environment. Root structures of WT plants are modified by the environments. The shoots of engineered plants are shown as “?”s to emphasize the idea that a specific root structure may affect plant health differently in different environments. B, Here, root structure is defined by a synthetic regulatory program and environmental stimuli cannot alter root growth. Shoot development is not engineered and will change in response to the water and nutrients obtained by the root system. Thus, the plant could be used to measure the root structure’s contribution to shoot health.

One compelling reason to engineer auxin-independent growth is to generate plants that can be used to probe the relationship between plant form and function. As stated above, plants respond to their environment by modifying their growth. Everything from nutrient abundance to water scarcity, commensal microbes to pathogens, and light quality to CO_2_ concentration can affect the plant growth (Fitter and Hay, 2002). Known relationships between plants’ form and fitness have inspired researchers to propose architectural ideotypes, that is, plant forms that should be ideal, for growth in different environments (Lynch, 2013, 2019; Schmidt and Gaudin, 2017). Unfortunately, testing ideotype hypotheses is difficult because mutations that alter plant form are typically pleiotropic, meaning they alter the development of multiple organs simultaneously (Guseman et al., 2017; Kuanar et al., 2019). Pleiotropic effects make it difficult to definitively attribute a change in survival to a specific plant organ. Plus, since plants also change their growth in response to the environment, the impact of a specific organ structure on plant fitness cannot be tested across multiple conditions. Thus, plants that grow into the same shape in all environments would allow researchers to measure a structure’s contribution to overall plant fitness (Figure 3, A). By enabling direct testing of plant form and function relationships, synthetic plant development could enhance our understanding of the mechanisms by which plants survive stress. The resulting information can then be used to design plant growth programs that are optimized for survival in a specific environment.

Reduced auxin sensitivity may lead to reduced environmental sensitivity. Many of the plants’ responses to environmental cues are mediated by hormones (Mazzoni-Putman et al., 2021). For example, abscisic acid (ABA) inhibits root and shoot elongation in response to drought and osmotic stress (Chen et al., 2020). Brassinosteroids (BR) promote stem growth in response to shade. Although these hormones alter plant growth, they do so largely by modifying auxin response (recently reviewed by Semeradova et al., 2020). ABA interferes with the trafficking of auxin transporter PIN2 to change root growth (Li et al., 2020) and BR modifies *IAA9* and *ARF7* expression during hypocotyl elongation (Zhou et al., 2013). Synthetic developmental programs that express recoded and mutated ARFs and Aux/IAAs might be less sensitive to these regulatory mechanisms. When plant development is engineered to be less sensitive to auxin, plants may develop more like animals; with post embryonic growth primarily defined by genetics, rather than the environment. (Figure 3, B, Outstanding Questions Box).

Although environment-insensitive growth could be useful for probing relationships between plant form and function, it will be challenging to implement. Hard physical limitations exist that may not be overcome by synthetic genetic regulation. For example, plant cells need water to expand and maintain turgor pressure, thus a large plant may not be physically possible to build in a completely dry environment (Scharwies and Dinneny, 2019). Plus, the activity of tissue-specific promoters used to drive the expression of synthetic developmental regulation may be altered by the environment and cause the programs to fail. Although the feasibility of environment-insensitive growth has its limits, the process of building auxin/environment-insensitive developmental programs will undoubtedly challenge and enhance our current understanding of plant development (Outstanding Questions Box).

## Expanding to nonmodel plants

One advantage of developing a framework for engineering plant form that is based on auxin response regulators is the potential ease of expansion into nonmodel plants. ARFs and Aux/IAAs are highly conserved across plant species, which makes them easy to identify in genomic sequences (Li et al., 2016; Wu et al., 2017). Where tested, ARFs and Aux/IAAs have been found to play important roles in the development of these plants. For example, rice Aux/IAA proteins OsIAA11 and OsIAA23 are critical for lateral root development and stem cell maintenance in rice (Jun et al., 2011; Zhu et al., 2012). Importantly, similarity in ARFs and Aux/IAAs from diverse plant species means that the mutations that make Arabidopsis ARFs and Aux/IAAs insensitive to auxin are likely to have the same effects on regulators from other plant species. Indeed auxin-desensitizing gain-of-function mutations in rice Aux/IAAs occur in the same domain as Arabidopsis’ Aux/IAAs (Zhu et al., 2012; Song and Xu, 2013). Thus, it should be reasonably straightforward to turn other plants’ ARFs and Aux/IAAs into auxin-insensitive tools for engineering structure. This conservation means ARFs and Aux/IAAs will be significantly easier to use for modifying development than other regulators, which may not have homologs with conserved function in the target species.

**Figure 4:**
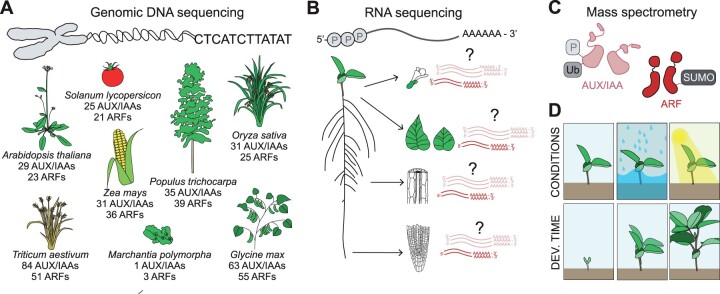
Leveraging -omics techniques for engineering beyond Arabidopsis. A, ARFs and Aux/IAAs identified in diverse plant species through genomic sequencing. *Source*: *Glycine max* (soybean) (Van Ha et al., 2013; Singh and Jain, 2015), *M. polymorpha* (Flores-Sandoval et al., 2015), *O. sativa* (rice) (Jain et al., 2006; Wang et al., 2007), *Populus trichocarpa* (Kalluri et al., 2007), *Solanum lycopersicon* (tomato) (Wu et al., 2011; Audran-Delalande et al., 2012), *T. aestivum* (wheat) (Qiao et al., 2015; Gidhi et al., 2020), *Z. mays* (maize) (Wang et al., 2010; Liu et al., 2011). B, Hypothetical single-cell RNA sequencing pipeline to identify cell types expressing ARF and Aux/IAA genes. C, Mass spectrometry to identify post-translational modifications on ARF and Aux/IAA proteins. D, Transcriptomics and proteomics analysis should be run across multiple environments and developmental stages to associate individual ARFs and Aux/IAAs with specific developmental processes.

Single-cell -omics techniques can be used to associate individual ARFs and Aux/IAAs with specific developmental processes in nonmodel plants. Most flowering plants contain large families of ARFs and Aux/IAAs with very similar sequences (Figure 4, A). Though helpful in identifying ARFs and Aux/IAAs in plant genomes, sequence similarity between family members means individual ARFs and Aux/IAAs cannot be associated with specific developmental pathways by gene sequence alone. Advances in single-cell -omics techniques in plants can help identify candidate ARFs and Aux/IAAs to mutagenize and express in order to manipulate a specific developmental process. Recent breakthroughs in RNA-sequencing technologies have enabled single-cell transcriptomics in plants (Rich-Griffin et al., 2020). When performed on Arabidopsis roots, scRNA-seq has identified genes involved in lateral root initiation (Gala et al., 2021) and endocycle maintenance (Torii et al., 2020). In other plant species, transcriptomics could be used to reveal the tissues and times in which ARFs and Aux/IAAs are expressed (Figure 4, B). Similarly, phosphoproteomics could identify ARFs and Aux/IAAs posttranslational modifications that could be either mimicked or eliminated to generate the developmental tools (Figure 4, C). Although no single-cell phosphoproteomic studies have been performed yet in plants, bulk phosphoproteomic analyses have identified phosphorylated proteins that are important for stress growth regulation (Bhaskara et al., 2017; Wong et al., 2019). Finally, experiments run across environments and developmental stages will further enhance our understanding of development in nonmodel plants and enable the engineering of their form (Figure 4, D).

## Conclusion

In a world where climate variables are predicted to change more rapidly than the natural rate of genetic adaptation, it is increasingly important to develop new methods for generating robust crop varieties. The auxin approach outlined in this review is only one of a growing number of strategies for engineering plant form. Recently, CRISPR-Cas9 editing has been used to selectively mutagenize the promoters of signaling peptides in order to increase fruit size in tomato (Rodríguez-Leal et al., 2017; Wang et al., 2021) and grain yield in maize (Liu et al., 2021). Hormone-sensitive synthetic transcription factors were created to alter shoot branching in Arabidopsis and plant size in tomato (Khakhar et al., 2018, 2021). A tissue-specific promoter was used to express a cytochrome P450 that modified maize leaf and ear structure without pleiotropic effects (Sun et al., 2017). It is my hope that all approaches will be used to generate robust crop varieties that enable agricultural stability in a dramatically changing climate.

**Advances**

- Molecular tools for controlling transcription and translation have been recently developed for plants that enable unprecedented control over gene expression.
- Decades of research into the molecular mechanisms underpinning plant development may enable genetic engineers to design synthetic regulatory programs that alter specific aspects of plant structure.
- Progress in refactoring complex phenotypes in prokaryotes suggests a refactoring-like approach may be useful for engineering complex processes, such as development, in plants.
- Single-cell sequencing and proteomics approaches are making it easier to study and potentially engineer nonmodel plants.

## Notes

J.A.N.B. conceived of and wrote this review.

The author responsible for distribution of materials integral to the findings presented in this article in accordance with the policy described in the Instructions for Authors (https://academic.oup.com/plphys/pages/general-instructions) is: Jennifer A.N. Brophy (jennbrophy@gmail.com).
